# Death from Eating Fruit

**Published:** 1838

**Authors:** R. C. Mann

**Affiliations:** Mount Pleasant, Hamilton County, Ohio


January 1838.

---

### Art. VII.—Death from eating Fruit.

*A case of Death apparently from eating unripe Apples, and the fruit of the* Prunus Virginiana, *or Wild Cherry.* By Dr. R. C. Mann, of Mount Pleasant, Hamilton County, Ohio.

On the 18th of August, 1837, I was requested to visit C. B., a strong, healthy boy, aged five years, and arrived at 4 o'clock P. M. when he presented the following symptoms:—Pulse about 160, small, quick, irregular, wiry; mouth open; tongue partially protruded, of a dark red, and dry on the apex and edges, coated with a dirty grey fur at the base; teeth partly coated with sordes; eyes half open; pupils dilated one half beyond their usual size; blood vessels of the eyes distended, the balls covered with a thick glutinous secretion rolled upwards, and insensible to light, or the approach of external objects; respiration difficult, stertorous, thirty in a minute; skin on the face of a deep shining red, with a few purplish spots; trunk covered with a bright red crescent-shaped eruption, extremities with diffused livid blotches; epigastrium distended, resisting, very hot; head hot, and thrown backward; limbs cold. In this situation he remained, after my arrival, about 15

minutes, when he began to murmur; and this increased till he talked rapidly; his enunciation being indistinct, guttural, and attended with great muscular exertion. This paroxysm lasted about ten minutes, subsiding gradually till he sunk again into coma.

In reply to my inquiries, I learned, that 48 hours previously, he had, in company with some other boys, visited a neighboring orchard, where he ate wild cherries and unripe apples. He returned before night, ate a hearty supper of fried potatoes, with bread and butter, and retired early to bed, apparently well. In the night he arose, went to his mother, and said he was sick. She gave him some water, and sent him back to bed, without further attention. In the morning, she found him "with a high fever, and so sleepy she could not keep him awake." She at once concluded he had an "attack of worms," and commenced feeding him "worm tea," (*Chenopodii anthel. infus.*) His stupor and fever increased continually; but she was not alarmed, being confident he would be relieved by discharging the worms, and therefore continued her treatment during the day. At night, she gave him a table-spoonful of ol. ricini, and towards morning repeated the dose. At 7 A. M. worse; no evacuation; gave him 12 grains of calomel and 5 of rhubarb, and continued the anthelmintic infusion. His disease, however, increased in violence; the parents became alarmed, and despatched a messenger for me; but I could not visit him till the hour specified above. From the retching, distension of the epigastrium, and other symptoms, I was induced to believe the stomach still retained irritating ingesta, and immediately administered, in repeated and liberal doses, an emetic of tartarized antimony and ipecacuanha. At half past five o'clock, no change in the symptoms. Emetic solution all taken, without a symptom of emesis. Ordered venesection, to which the parents objected, declaring they "never knew any person get well, who was bled in so high a fever!" Substituted the cold dash freely, from a height of three feet. Pulse immediately sunk to 140, and became stronger and fuller. No sensorial relief. In twenty-five minutes, pulse rose to 165. Disregarding the fears of the parents, I now abstracted ten ounces of blood from the arm, which reduced the pulse to 120, and rendered it small, soft and compressible; but the sensorium was not in the least relieved. At 7 o'clock, pulse

170, irregular; all the symptoms worse; no evacuations. Ordered ol. crot. tig. gt. iv., in six pills; one to be given every forty minutes, till catharsis should commence, aided by frequent stimulating enemata; drink, alkaline. At 3 A. M., pills all taken. They produced four copious dejections, not seen by me, but described as green, undigested, and very offensive. Pulse 180, irregular and small; cutaneous eruption of a darker hue; hands and wrists quite purple; respiration 24, more laborious; eyes fixed and agglutinated; extremities stiff and cold; chest and head very hot. Continued cold applications to the head, and hot applications to the limbs. No medicine. The alternation of jactitation and coma had nearly subsided, and he remained wholly insensible, with occasional struggles, till 8 o'clock A. M., at which time he died, very much swollen in the abdomen.

*Sectiv cadaveris*, 8 hours after death. *External appearance.*—Tumefaction subsided; petechiæ livid on the limbs, sub-livid on the trunk and face; arms flexed and stiff. *Thorax.*—Lungs healthy; heart natural; pericardium containing three ounces of serum. *Abdomen.*—Stomach containing a little dirty greenish fluid; mucous membrane folded into large soft rugæ; cardiac portion in a state of inflammatory hyperæmia; pyloric region covered with dark gangrenous spots, in many of them the structure entirely destroyed; pylorus thickened, softened, gangrenous. *Duodenum.*—Mucous membrane softened; many livid spots; jejunum and ilium in a state of partial inflammation, occasional large red spots; cæcum and colon natural; three lumbricoides were found in the former; omentum thickened and gangrenous near the duodenum and small lobe of the liver; remainder of the peritoneum sound. *Liver.*—Structure sound; blood vessels full. Gall bladder distended with dark vitiated bile. Kidneys natural. Urinary bladder distended with yellow offensive urine. The other abdominal organs in a healthy state.

January 1838.

—

### Editorial Remarks.

Dr. Mann, in a note appended to his paper, states that no eruptive disease prevailed in the neighborhood of Mount Pleasant when this case occurred; otherwise it might be regarded as irregular measles or scarlatina. The comrades of his little patient affirmed, that he did not eat a large quantity of cherries; but the Doctor, very properly asks the question, whether the Hydrocyanic acid contained in them, was not a chief agent in the production of the disease. We are strongly inclined to answer in the affirmative, from having long known, that the fruit of our wild cherry tree is often productive of very serious effects, especially when the stones are swallowed with the pulp. There is then a combined action of an astringent principle producing constipation, a narcotic poison, and a mechanical irritant. In the case before us, there was the additional influence of a mass of unripe and ascerb apples, and a quantity of ordinary food. It is not surprising that such causes should awaken mucous inflammation, and fatal sympathetic cerebral irritation. Had the patient been vomited freely when he first began to complain, his life would probably have been preserved. D.

